# Supplementary material for: Integrative Spatial Proteomics and Single-Cell RNA Sequencing Unveil Molecular Complexity in Rheumatoid Arthritis for Novel Therapeutic Targeting
Source: Proteomes. 2025 May 22;13(2):17. doi: 10.3390/proteomes13020017 (PMC12196869; doi:10.3390/proteomes13020017)
Supplement: Supplementary file 1 [file proteomes-13-00017-s001.zip › Supplemetary figures 20250514.pdf]

Figure S1. Quantified proteins, peptides and protein sequence coverage of synovium LCMs with varied area sizes

A

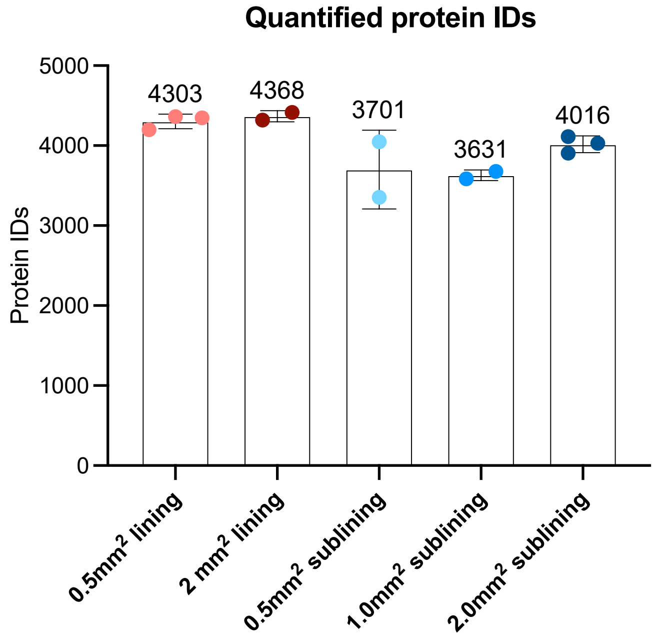

B

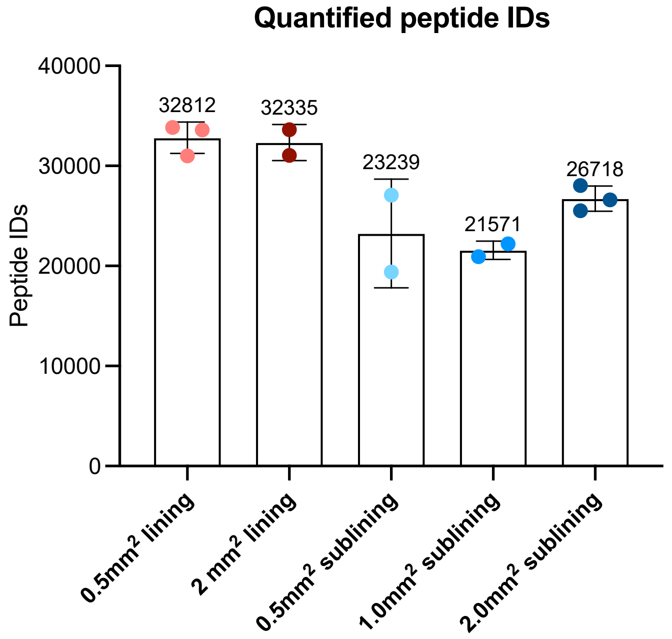

C

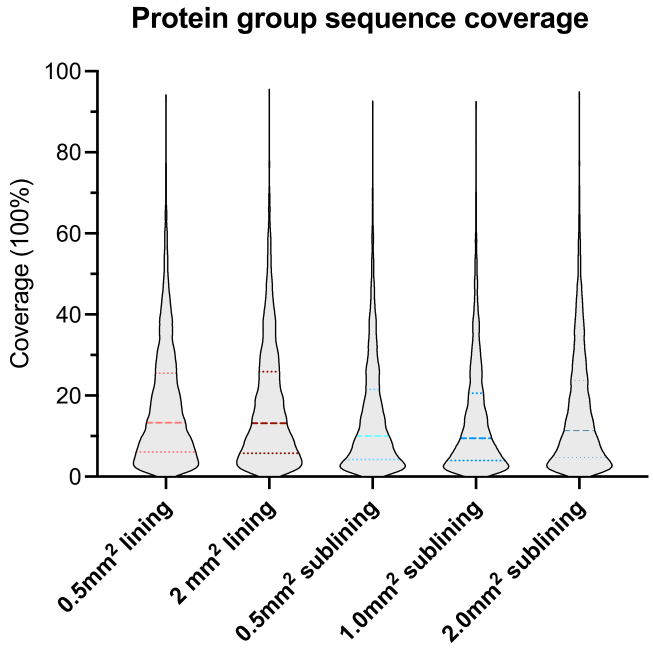

Figure S2. Quantified proteins and peptide ID numbers among region samples

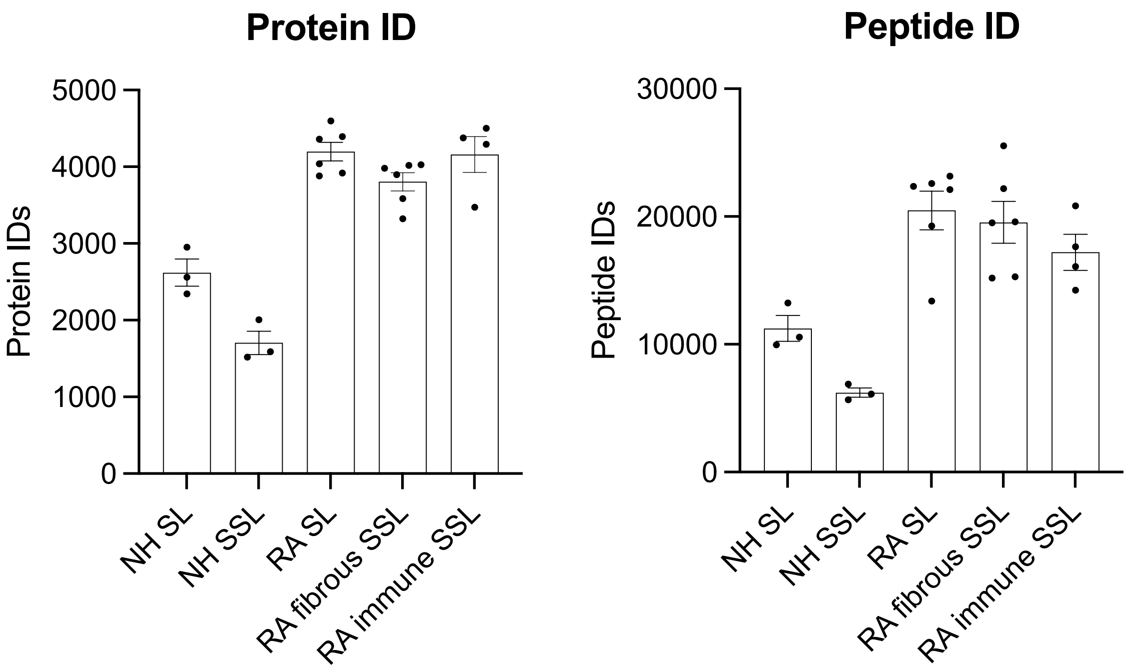

Figure S3. Proteins with maximum PC1 and PC2 loadings from PCA in 7 pairwise region comparisons overlap with differentially expressed proteins

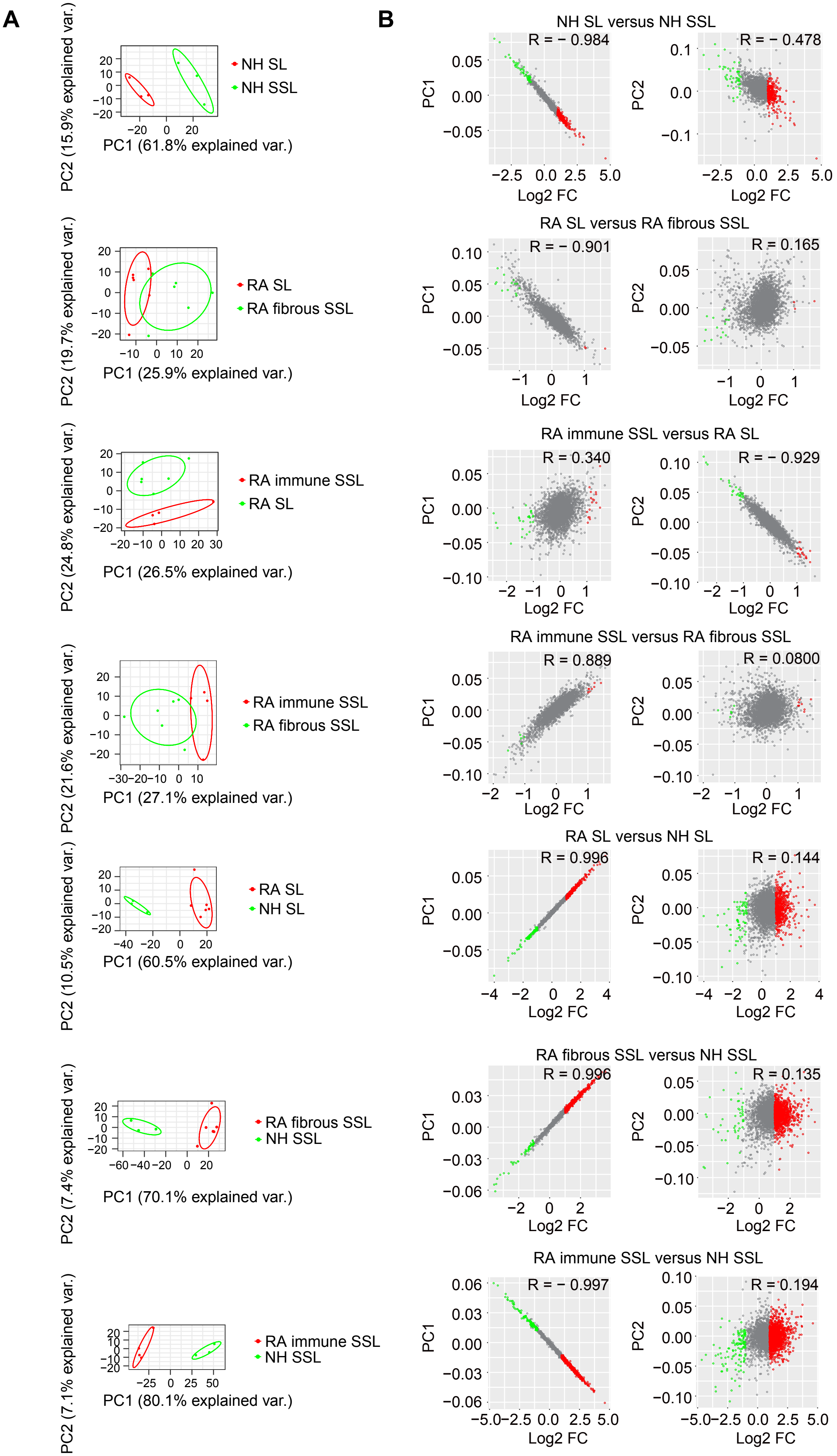

Figure S4. GSEA GO BP summary bubble plotting from protein differential expression (Log2 FC) in respective pairwise comparisons

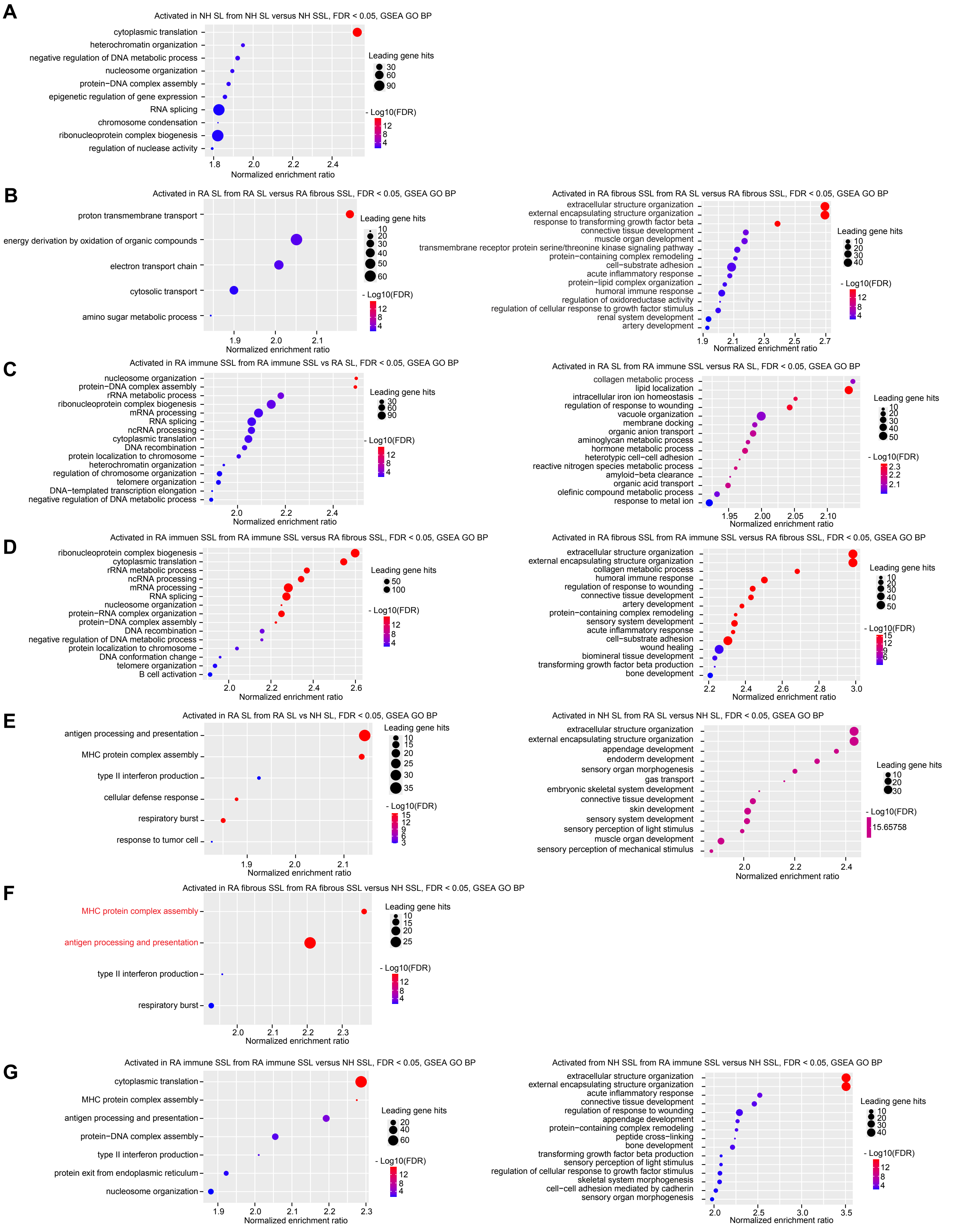

Figure S5. Cell subcluster depletions in different pathological compartments by the Query method integrating LCM spatial proteomics and scRNA-seq transcriptomics

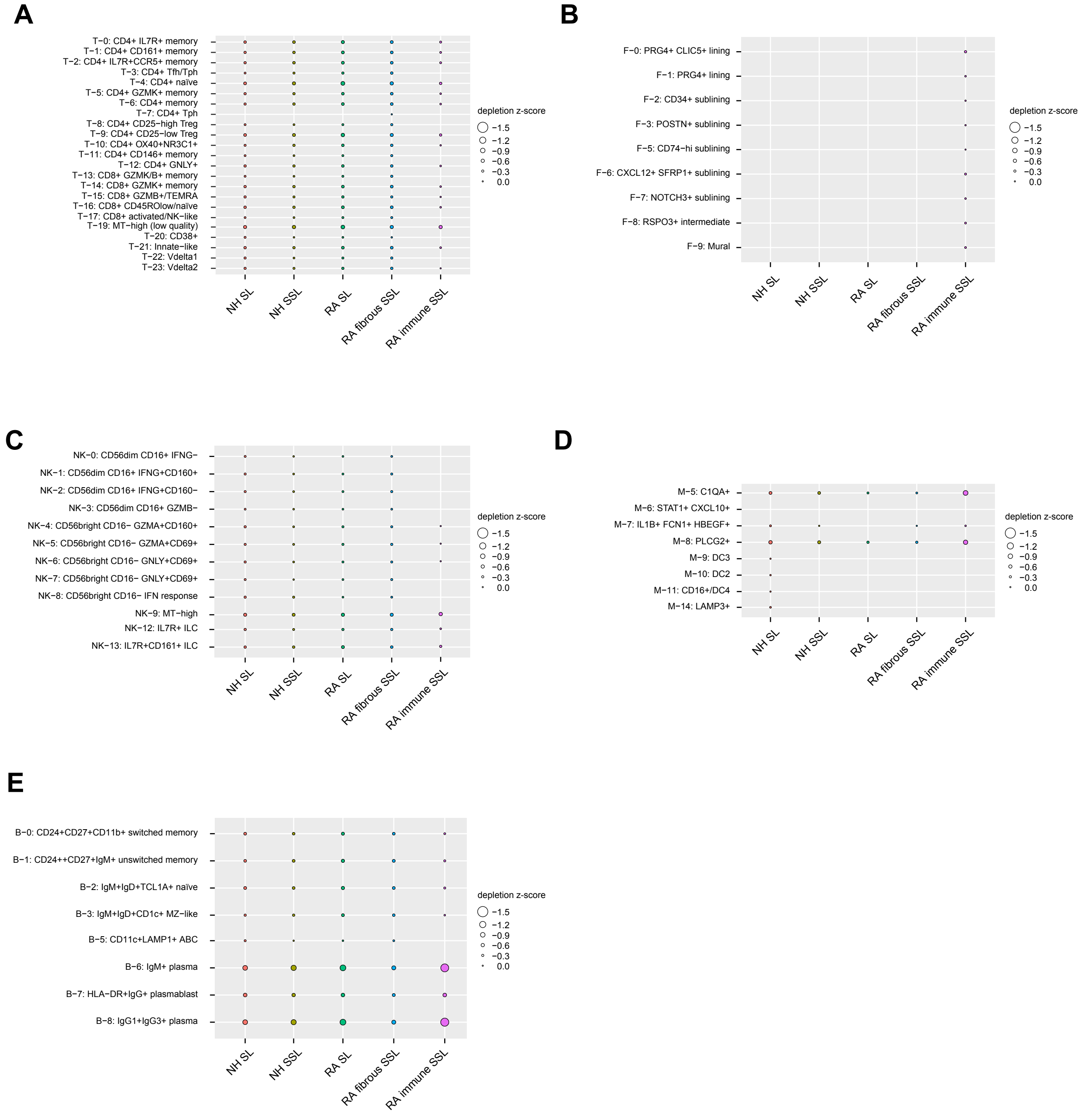

Figure S6: Example MS spectra of several peptides from FN1 and TCIRG1

A

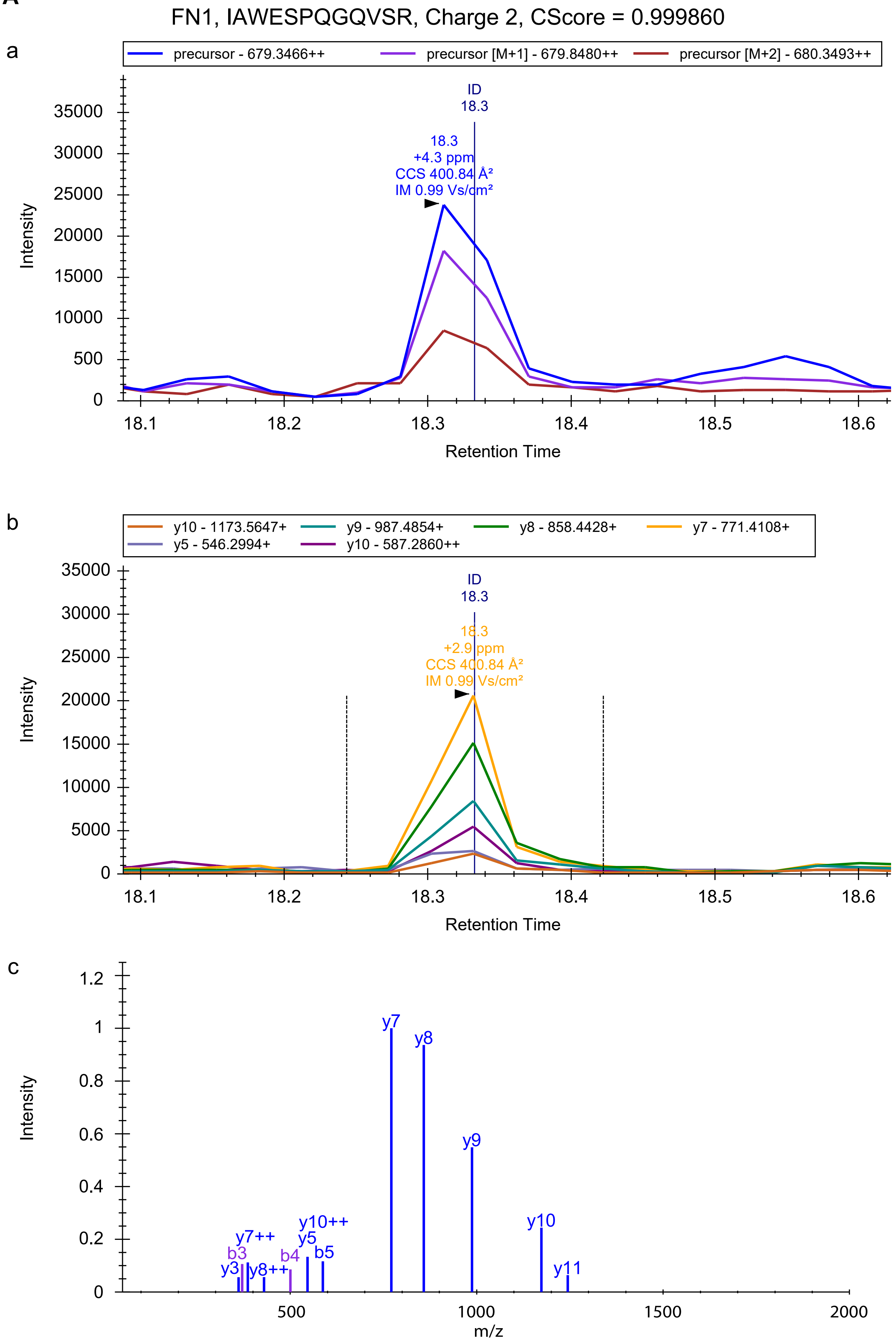

B

FN1, GLAFTDVDVDSIK, Charge 2, CScore = 0.999374

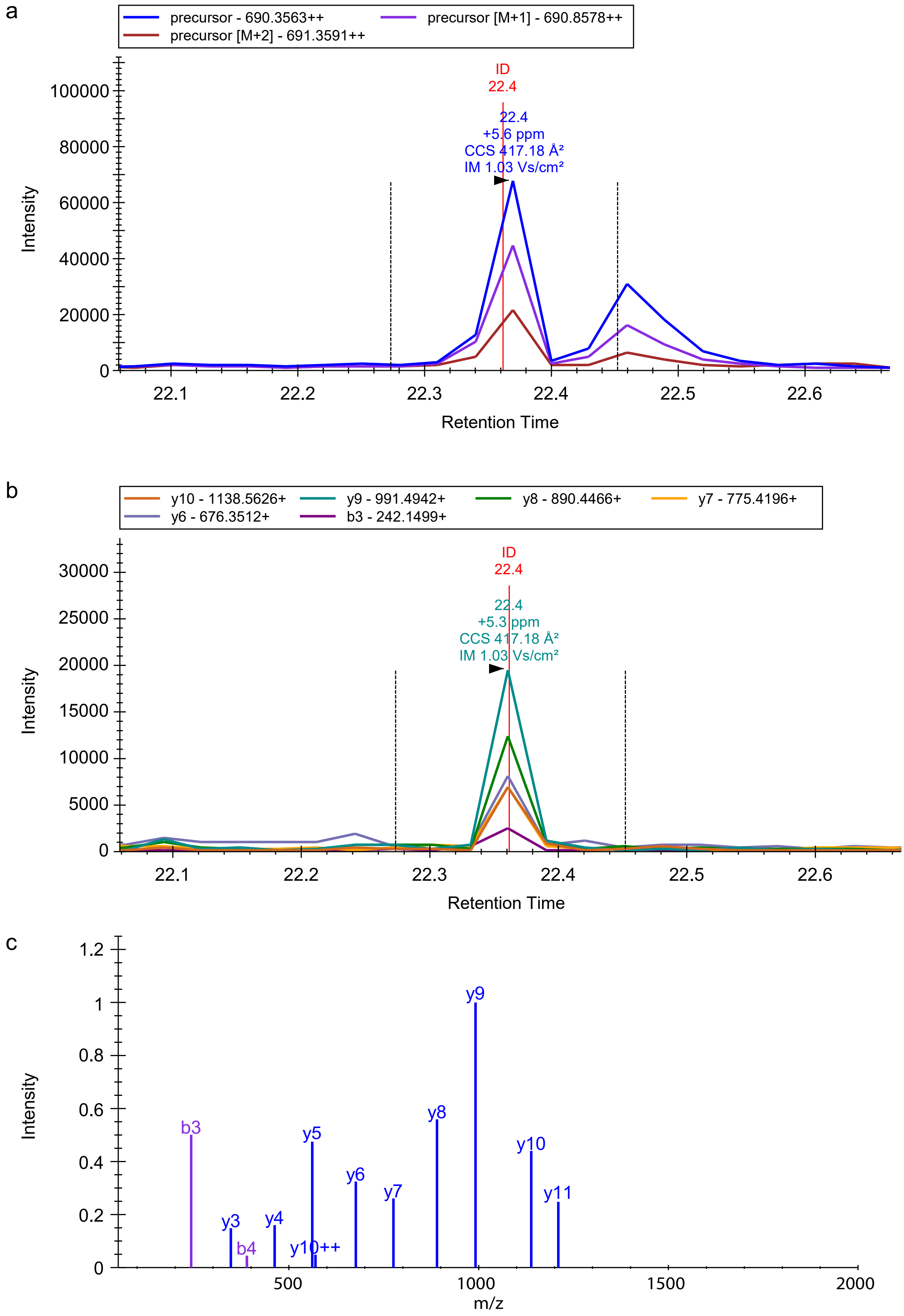

c

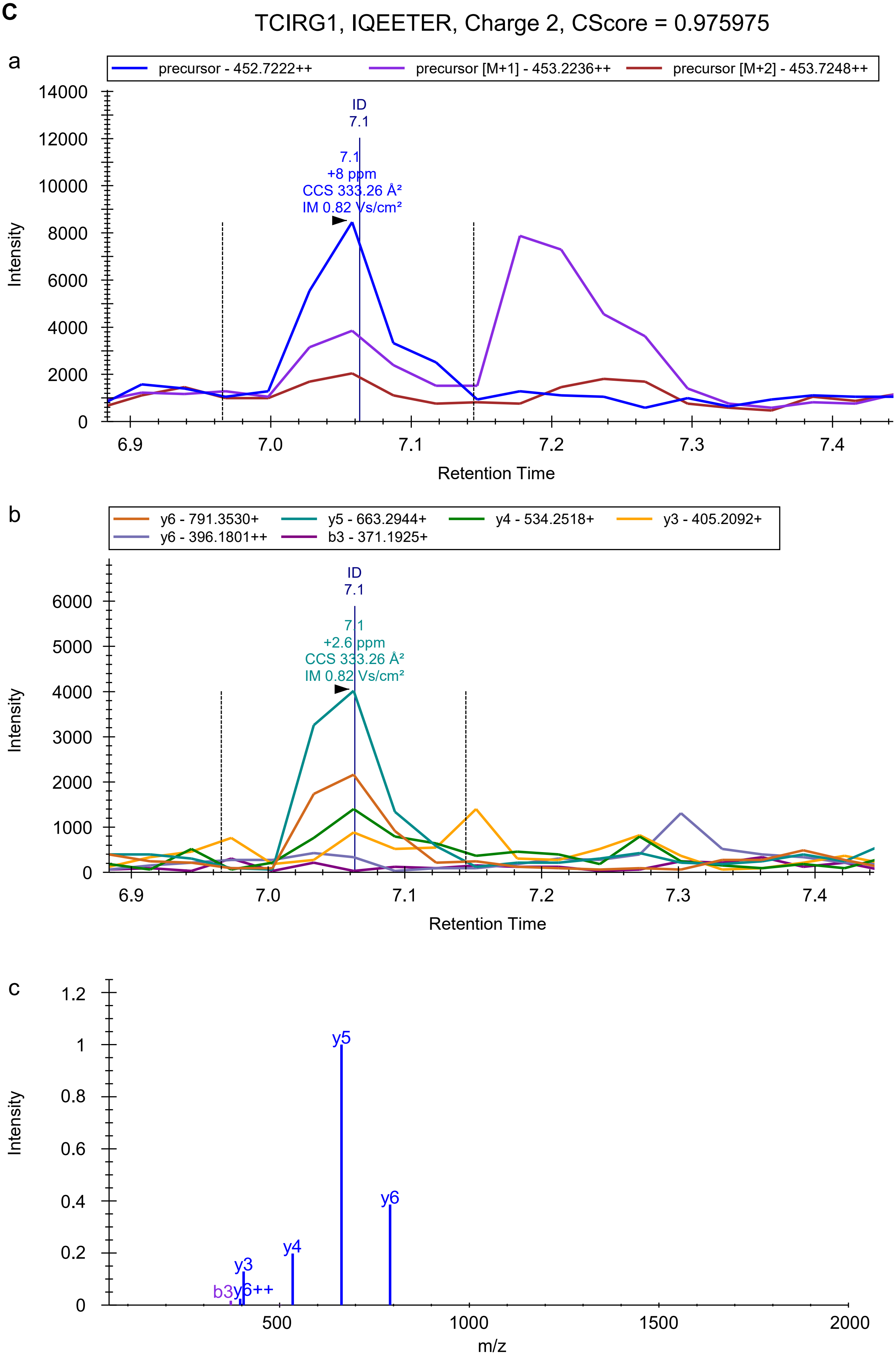
TCIRG1, IQEETER, Charge 2, CScore = 0.975975
a
precursor - 452.7222++
precursor [M+1] - 453.2236++
precursor [M+2] - 453.7248++
ID
7.1
7.1
+8 ppm
CCS 333.26 Å²
IM 0.82 Vs/cm²
Intensity
Retention Time
b
y6 - 791.3530+
y5 - 663.2944+
y4 - 534.2518+
y3 - 405.2092+
y6 - 396.1801++
b3 - 371.1925+
ID
7.1
7.1
+2.6 ppm
CCS 333.26 Å²
IM 0.82 Vs/cm²
Intensity
Retention Time
c
y5
y6
y4
y3
b3
y6++
Intensity
m/z

D

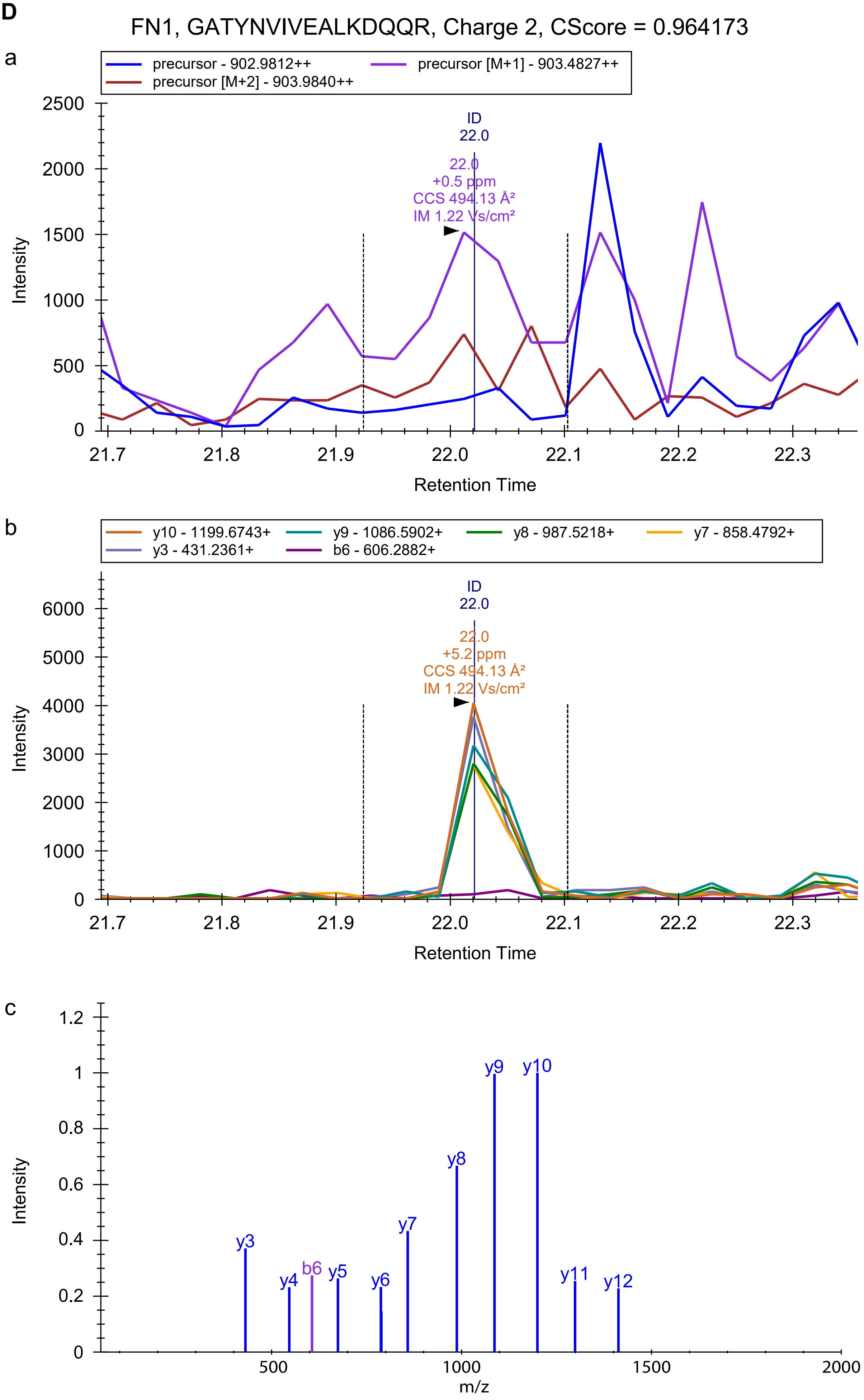

FN1, GATYNVIVEALKDQQR, Charge 2, CScore = 0.964173
a
precursor - 902.9812++
precursor [M+1] - 903.4827++
precursor [M+2] - 903.9840++
ID
22.0
22.0
+0.5 ppm
CCS 494.13 Å²
IM 1.22 Vs/cm²
Intensity
Retention Time
b
y10 - 1199.6743+
y9 - 1086.5902+
y8 - 987.5218+
y7 - 858.4792+
y3 - 431.2361+
b6 - 606.2882+
ID
22.0
22.0
+5.2 ppm
CCS 494.13 Å²
IM 1.22 Vs/cm²
Intensity
Retention Time
c
y3
y4
b6
y5
y6
y7
y8
y9
y10
y11
y12
Intensity
m/z

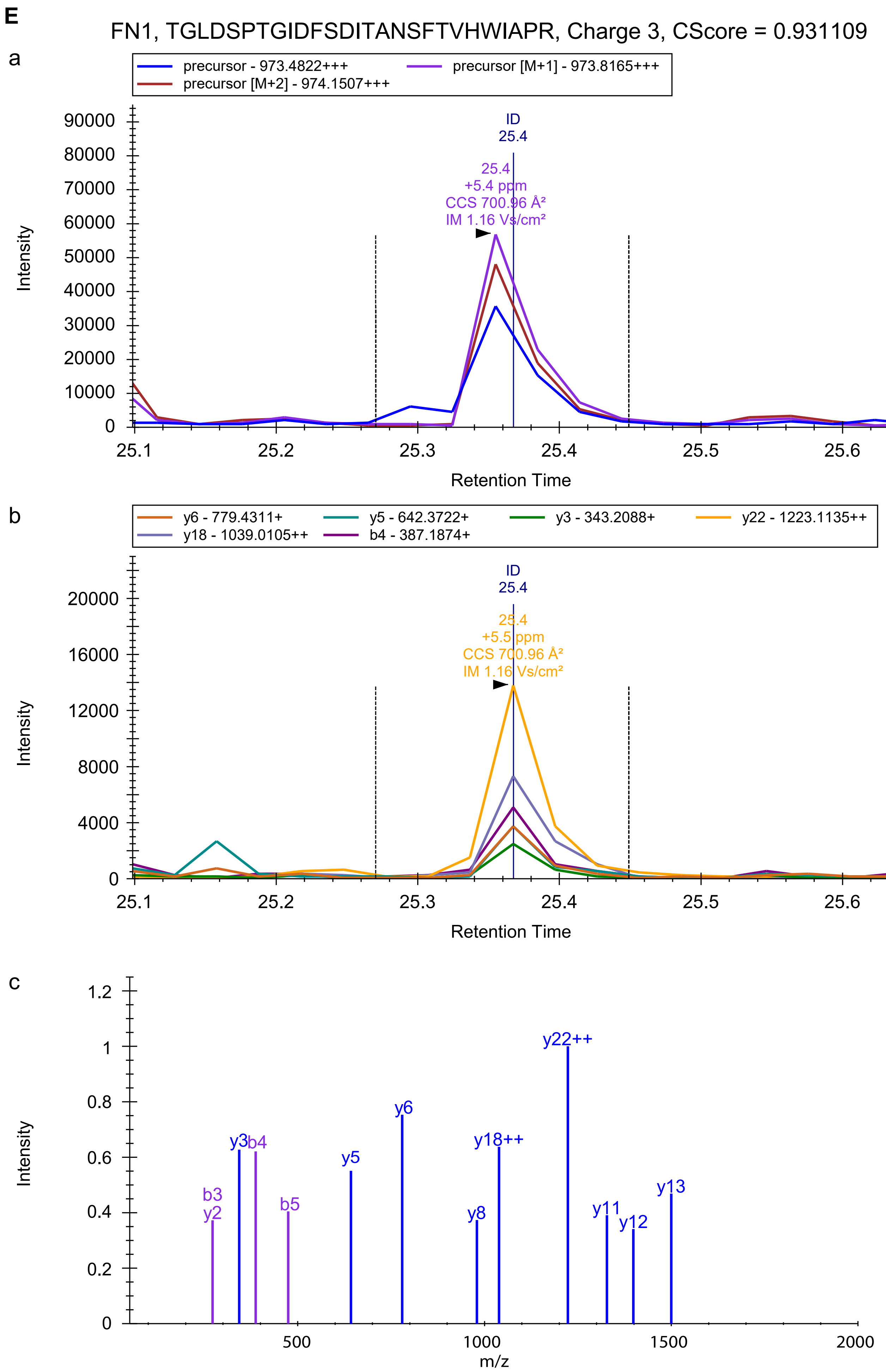
E
FN1, TGLDSPTGIDFSDITANSFTVHWIAPR, Charge 3, CScore = 0.931109
a
precursor - 973.4822+++
precursor [M+1] - 973.8165+++
precursor [M+2] - 974.1507+++
ID
25.4
25.4
+5.4 ppm
CCS 700.96 Å²
IM 1.16 Vs/cm²
Intensity
Retention Time
b
y6 - 779.4311+
y5 - 642.3722+
y3 - 343.2088+
y22 - 1223.1135++
y18 - 1039.0105++
b4 - 387.1874+
ID
25.4
25.4
+5.5 ppm
CCS 700.96 Å²
IM 1.16 Vs/cm²
Intensity
Retention Time
c
y22++
y6
y3
b4
y18++
y5
y13
b3
y2
b5
y8
y11
y12
Intensity
m/z

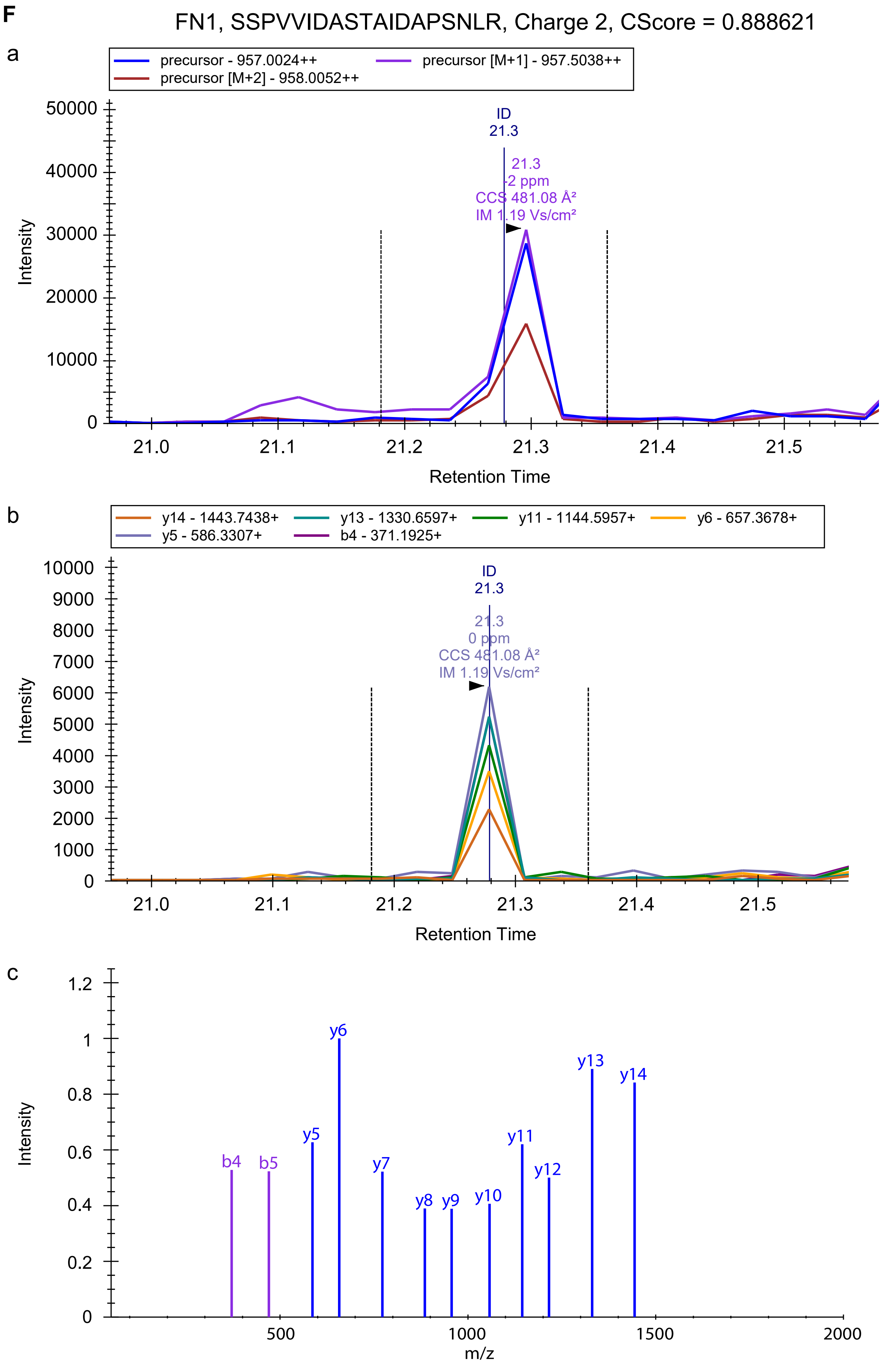
F
FN1, SSPVVIDASTAIDAPSNLR, Charge 2, CScore = 0.888621
a
precursor - 957.0024++
precursor [M+1] - 957.5038++
precursor [M+2] - 958.0052++
ID
21.3
21.3
-2 ppm
CCS 481.08 Å²
IM 1.19 Vs/cm²
Intensity
Retention Time
b
y14 - 1443.7438+
y13 - 1330.6597+
y11 - 1144.5957+
y6 - 657.3678+
y5 - 586.3307+
b4 - 371.1925+
ID
21.3
21.3
0 ppm
CCS 481.08 Å²
IM 1.19 Vs/cm²
Intensity
Retention Time
c
Intensity
b4
b5
y5
y6
y7
y8
y9
y10
y11
y12
y13
y14
m/z

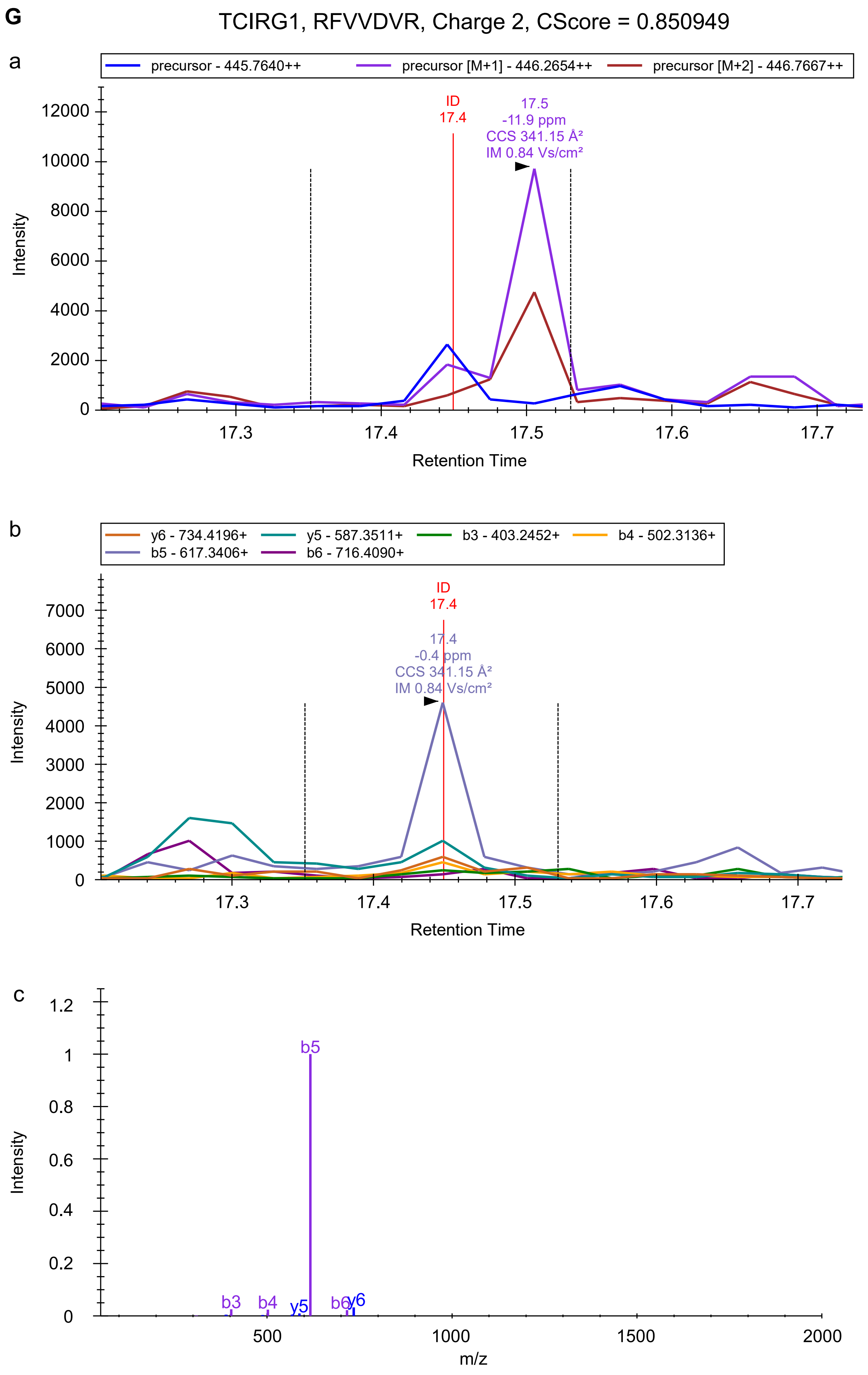
G
TCIRG1, RFVVDVR, Charge 2, CScore = 0.850949
a
precursor - 445.7640++
precursor [M+1] - 446.2654++
precursor [M+2] - 446.7667++
ID
17.4
17.5
-11.9 ppm
CCS 341.15 Å²
IM 0.84 Vs/cm²
Intensity
Retention Time
b
y6 - 734.4196+
y5 - 587.3511+
b3 - 403.2452+
b4 - 502.3136+
b5 - 617.3406+
b6 - 716.4090+
ID
17.4
17.4
-0.4 ppm
CCS 341.15 Å²
IM 0.84 Vs/cm²
Intensity
Retention Time
c
b5
b3
b4
y5
b6
y6
Intensity
m/z
